# Huvariome: a web server resource of whole genome next-generation sequencing allelic frequencies to aid in pathological candidate gene selection

**DOI:** 10.1186/2043-9113-2-19

**Published:** 2012-11-19

**Authors:** Andrew Stubbs, Elizabeth A McClellan, Sebastiaan Horsman, Saskia D Hiltemann, Ivo Palli, Stephan Nouwens, Anton HJ Koning, Frits Hoogland, Joke Reumers, Daphne Heijsman, Sigrid Swagemakers, Andreas Kremer, Jules Meijerink, Diether Lambrechts, Peter J van der Spek

**Affiliations:** 1 Department of Bioinformatics, Erasmus University Medical Center, Molewaterplein 50, Rotterdam, The Netherlands; 2 Department of Urology, Erasmus University Medical Center, Molewaterplein 50, Rotterdam, The Netherlands; 3 VX Company IT Services, Baarnsche dijk 8, 3741, LR, Baarn, The Netherlands; 4 Vesalius Research Center, VIB and University of Leuven, Gasthuisberg Herestraat 49, 3000, Leuven, Belgium; 5 Department of Pediatric Oncology, Sophia Children’s Hospital, Erasmus University Medical Center, Molewaterplein 50, Rotterdam, The Netherlands

**Keywords:** Medical genetics, Medical genomics, Whole genome sequencing, Allele frequency, Cardiomyopathy

## Abstract

**Background:**

Next generation sequencing provides clinical research scientists with direct read out of innumerable variants, including personal, pathological and common benign variants. The aim of resequencing studies is to determine the candidate pathogenic variants from individual genomes, or from family-based or tumor/normal genome comparisons. Whilst the use of appropriate controls within the experimental design will minimize the number of false positive variations selected, this number can be reduced further with the use of high quality whole genome reference data to minimize false positives variants prior to candidate gene selection. In addition the use of platform related sequencing error models can help in the recovery of ambiguous genotypes from lower coverage data.

**Description:**

We have developed a whole genome database of human genetic variations, Huvariome, determined by whole genome deep sequencing data with high coverage and low error rates. The database was designed to be sequencing technology independent but is currently populated with 165 individual whole genomes consisting of small pedigrees and matched tumor/normal samples sequenced with the Complete Genomics sequencing platform. Common variants have been determined for a Benelux population cohort and represented as genotypes alongside the results of two sets of control data (73 of the 165 genomes), Huvariome Core which comprises 31 healthy individuals from the Benelux region, and Diversity Panel consisting of 46 healthy individuals representing 10 different populations and 21 samples in three Pedigrees. Users can query the database by gene or position via a web interface and the results are displayed as the frequency of the variations as detected in the datasets. We demonstrate that Huvariome can provide accurate reference allele frequencies to disambiguate sequencing inconsistencies produced in resequencing experiments. Huvariome has been used to support the selection of candidate cardiomyopathy related genes which have a homozygous genotype in the reference cohorts. This database allows the users to see which selected variants are common variants (> 5% minor allele frequency) in the Huvariome core samples, thus aiding in the selection of potentially pathogenic variants by filtering out common variants that are not listed in one of the other public genomic variation databases. The no-call rate and the accuracy of allele calling in Huvariome provides the user with the possibility of identifying platform dependent errors associated with specific regions of the human genome.

**Conclusion:**

Huvariome is a simple to use resource for validation of resequencing results obtained by NGS experiments. The high sequence coverage and low error rates provide scientists with the ability to remove false positive results from pedigree studies. Results are returned via a web interface that displays location-based genetic variation frequency, impact on protein function, association with known genetic variations and a quality score of the variation base derived from Huvariome Core and the Diversity Panel data. These results may be used to identify and prioritize rare variants that, for example, might be disease relevant. In testing the accuracy of the Huvariome database, alleles of a selection of ambiguously called coding single nucleotide variants were successfully predicted in all cases. Data protection of individuals is ensured by restricted access to patient derived genomes from the host institution which is relevant for future molecular diagnostics.

## Background

Next-generation sequencing (NGS) provides scientists with the ability to screen for genetic variants at a higher density than genome wide screens with array based platforms [1]. The choice of sequencing the whole genome or only the exome, the latter comprising approximately 1% of the entire genome, depends on the type of research question to be addressed. Exome sequencing delivers information on the coding regions of the genome [2] and has been successfully applied, and continues to be applied, to determine the causative genetic event in Mendelian inherited diseases [3]. Whole genome sequencing provides scientists with an unbiased view of genetic variation of the genome including promoters, intronic splicing regulators, regulatory regions (enhancers, silencers), non-coding RNAs (microRNAs, snoRNAs, lincRNA) and structural variation including copy number [4]. There are 3 to 4 million SNVs per human genome of which approximately 10% are novel variants, some of which are false positives and may confound the selection of disease causing variants [4]. Variants detected in other genomes are less likely to be artifacts; hence the use of databases to store high quality personal variants will improve the detection of pathogenic variants. The advent of whole genome and exome sequencing tests, replacing the single variant assay as clinical genetics tests and for cancer diagnosis based on reduced costs, will require access to large scale central databases to distinguish clinically relevant variations from neutral polymorphisms [5]. The central requirement for implementing NGS into clinical practice is to allow simple and secure access to databases containing curated knowledge of variants scored as clinically relevant pathogenic mutations with standardized clinical reporting. Several existing projects that support the detection of common deleterious variants in the population include Online Mendelian Inheritance in Man [6], dbSNP [7], Database of Genomic Variants [8] and Human Gene Mutation Database [9]. SeattleSeq Annotation [10] and ENGINES [11] are both web services for easy access to the genotypes stored in dbSNP, and for annotation of variants for both hg18 and hg19 genome builds. NGS catalog [12] which is built on SeattleSeq provides scientists with an integrated view of public literature derived variation results, summarized by sequencing platform type (e.g. RNAseq), technology platform (e.g. HiSeq2000) and linked to the publication from which the results were derived. ANNOVAR [13], a command line tool, is popular with bioinformaticians and is used to annotate experimentally derived variants with common and rare variants derived from the popular sources (e.g. dbSNP), the 1000 genomes project [14], and Exome Variant Server [15], and to provide functional impact where appropriate in coding regions. However, to the medical research scientist the majority of these results have been made available in the web application SNPnexus [16], which delivers functional annotation of novel and known variants and improved access via positional mapping through contig or clone coordinates. Huvariome provides the user with whole genome allele frequencies, their associated quality score (detection and chance to detect the variant), gene based ranking and integrated access to publicly available data for the detection of common, rare and deleterious variants. The functional impact of variants in Huvariome is provided by the Complete Genomics (CG) annotation pipeline [17]. The novelty of Huvariome is that it provides rapid and simple access to SNV, short indels, and *de novo* assembled regions of the genome at any position in the genome with allelic frequencies and associated error for position in the human genome. Huvariome also delivers common variants from a small cohort of Benelux genomes from unrelated individuals with no disease association. In light of these developments we have developed a simple application, Huvariome, which goes beyond the current platforms with similar goals [10,11] to enable efficient allelic frequencies searching in both public and private genomes for clinical research scientists.

## Construction and content

### Subjects

#### Standard for human subject and data protection

All records for the biological specimens are maintained within the hospital health record management system and an anonymized sample code was supplied with the DNA and used to map the returned sequence data to the appropriate sample information stored in the database. All subjects whose whole genome sequence (WGS) results are stored within the database were approved of by the Institutional Review Board of the Erasmus MC, Rotterdam, the Netherlands (MEC-2011-253, date of approval February 27^th^, 2011) in which patients gave written informed consent according to institutional and national guidelines. Formalized meta-data relating to the individual from whom the genome was sequenced, but with no name or hospital identification code, is stored (Table 1), thus preventing the individual from being identified from the database. In addition the variants within a genome for the samples in Huvariome which are not publicly available are not presented on the public user interface to ensure that these individuals cannot be identified by their genomic variation.

**Table 1:** Standardized parameters for meta-data

**Parameter**	**Description**	**Limit**
Age	Age	Variable
Gender	Male , Female, other	Fixed
Ethnicity	HapMap groups	Fixed
Country of origin (if known)	UK, Netherlands, …	Variable
Study Type	Cancer, Family, Reference	Fixed
Biomaterial	Blood, Tissue, Cell line	Fixed
Biomaterial Subtype	PBMC, WBC, Heart, B-cell, …	Variable
Biomaterial Source	Peripheral vien, …	Variable
Biomaterial Modification	EBV transformed, …	Variable

The Description defines the options that are available for each Parameter. A parameter for which the Limit is fixed means that there are a fixed number of possible values whereas one that is Variable is stored as any value (free text or numeric).

### Next generation sequencing

Paired-end sequencing for all DNA samples was performed with the Complete Genomics service provider using a proprietary sequencing-by-ligation technology [17]. Complete Genomics also performed primary data analysis, including image analysis, base calling, alignment and variant calling. Reads were mapped to the NCBI Build 36.1 reference genome using a fast algorithm and initial mappings were expanded by local *de novo* assembly on all regions of the genome that contain single nucleotide variations (SNVs) relative to the reference genome [17]. Sequencing reads were mapped to the reference genome with versions 1.1.0 to 1.12.0 of the Complete Genomics Analysis (CGA) pipeline from which the derived variant files include the SNVs, inserts and deletions (indels), and substitutions (subs) with confidence scores and explicit differentiation of “no-variant” from “no-call”. Currently these data are shipped as bzip format on 1.5 Tb discs and uploaded to the Department of Bioinformatics IBM server. The resultant genomes are at least 40X (~120 GB) mapped coverage with accurate calls >95% for the genomes.

## Development of the database

### Informatics infrastructure

Huvariome is developed with an Oracle 11i 64-bit relational database (Enterprise Edition Release 11.2.0.1.0), the code developed in Perl 5.8.8 and the graphical user interface developed with PHP 5.3.3, and is available on an Apache 2.2.3 server. The database is designed to store all variation types detected by Complete Genomics and are supplied in the variation results file (“VAR” file) which includes SNVs, indels and subs up to ~100bp as defined by the Complete Genomics Release Notes Assembly Software v2.0 [18]. Those variations that occur in a gene (5′UTR, 3′UTR, exon and intron) are supplied with additional annotation describing the associated gene in the gene file. The VAR and gene files are loaded into the Oracle database using a custom loader that was developed to provide quality assurance upon upload and to be easily adapted to accommodate changes in the annotation pipeline of Complete Genomics. The database stores variants relative to a reference genome such that only differences to the reference are listed, allowing for a substantial reduction of data. Each genome is annotated with a minimal set of required information detailing the individual sample and the relationship with other samples (e.g. tumor versus paired normal and parents versus children). This minimal information of the genome source ensures that the propensity of variation to appear in a subset of the data can be traced and allows users to perform meta-analyses across the whole database to rapidly identify cancer associated and family based variants. Currently all genomes are mapped to NCBI build 36.3 and annotated using RefSeq data for gene and protein annotations, dbSNP version 130 [7], DGV [8] for known variations and GenomeTrax^TM^ (Biobase, Germany) for multiple annotations including HGMD Professional.

#### Development of the schema

**Figure 1:**
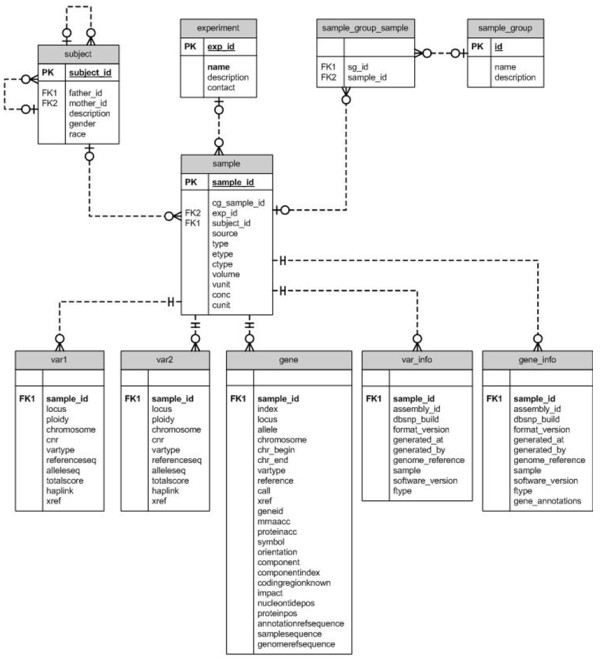
**Huvariome database schema.** The part of the database schema representing the relationship between the core variation and annotation tables based on the content delivered by Complete Genomics.

Database design was developed to reflect, where possible, the original data tables supplied by Complete Genomics [18] to ensure the ability to store genomes as the data output from the CGA pipeline advances with richer annotation and improved quality measurements included with the supplied data. Variation data, SNVs, indels and subs, supplied in the VAR files are stored as alleles in the var Tables 1 and 2 (Figure 1). Annotation can be scaled to include any annotation type or source including the reference genome associated with the original mapped reads from the primary sequencing files. Annotation is connected to allow for fast updates and migration, e.g. to NCBI build 37 and meta-data concerning the individual genomes, and the minimal information defining a phenotype is stored in a sample table (Figure 1).

**Table 2:** Summary of huvariome genomes

**Description**	**Female (range)**	**Male (range)**	**All (range)**
Gross mapping yield (Gb)	206 (160–280)	202 (168–249)	206 (160–280)
Fully called genome fraction	95.13% (91%/97%)	95.89% (94%/97%)	95.13% (91%/97%)
Partially called genome fraction	0.96% (0%/2%)	0.69% (0%/1%)	0.96% (0%/2%)
No-called genome fraction	3.91% (3%/7%)	3.42% (2%/5%)	3.91% (2%/7%)
SNP total count	3257897 (2966002–3396520)	3275018 (3056972–3495143)	3257897 (2966002–3495143)
SNP novel rate	6.71% (6%/7%)	6.78% (6%/9%)	6.71% (6%/9%)
Synonymous SNP	9239 (8668–9564)	9229 (8503–9821)	9239 (8503–9821)
Missense SNP	9046 (8348–9456)	9037 (8380–9574)	9046 (8348–9574)
Nonsense SNP	96 (86–117)	95 (77–110)	96 (77–117)
Nonstop SNP	24 (19–29)	22 (17–26)	24 (17–29)
INS total count	180040 (152082–208451)	190177 (160473–209226)	180040 (152082–209226)
INS novel rate	21.31% (19%/23%)	21.58% (19%/23%)	21.31% (19%/23%)
Frame-shifting INS	134 (113–157)	130 (95–152)	134 (95–157)
Frame-preserving INS	116 (96–132)	117 (98–130)	116 (96–132)
DEL total count	192550 (157937–217782)	202085 (166805–227228)	192550 (157937–227228)
DEL novel rate	23.85% (23%/25%)	23.75% (22%/26%)	23.85% (22%/26%)
Frame-shifting DEL	117 (96–144)	110 (86–126)	117 (86–144)
Frame-preserving DEL	120 (100–138)	115 (106–128)	120 (100–138)
SUB total count	68020 (56179–75040)	69396 (59319–76699)	68020 (56179–76699)
SUB novel rate	34.07% (31%/38%)	33.96% (31%/37%)	34.07% (31%/38%)
Frame-shifting SUB	21 (11–27)	19 (14–26)	21 (11–27)
Frame-preserving SUB	259 (208–320)	252 (225–279)	259 (208–320)

The data represent the average counts from the 31 genomes of Huvariome Core in which the fraction of heterozygous SNPs, inserts, deletions or substitutions are not found in dbSNP (SNP, INS, DEL, SUB novel). The number of loci where a coding SNV did not result in protein sequence change (Synonymous SNP), number of loci where a coding SNV resulted in protein sequence change, with no change in size of protein (Missense SNP), number of loci where the single nucleotide change in coding sequence resulted in a STOP codon (TGA, TAG, or TAA), causing an early termination of protein translation (Nonsense SNP), number of loci where the single nucleotide change in coding sequence resulted in the change of a STOP codon into a codon that codes for an amino acid, resulting in the continuation of the translation for this protein (Nonstop SNP), number of loci where the single nucleotide change in coding sequence resulted in the change of a START codon into a codon for something other than a start codon, likely resulting in a non-functional gene (Misstart SNP). The number of insertion, deletion or substitution loci where the change in coding sequence resulted in a frameshift for the encoded protein (Frame-shifting INS, DEL, SUB), number loci where there is a change in coding sequence and the length of the insertion is a multiple of 3, resulting in the insertion of amino acids in the encoded protein in-frame (Frame-preserving INS, DEL, SUB).

#### User interface

**Figure 2:**
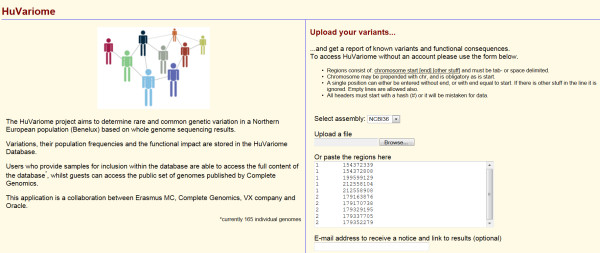
**Huvariome database access screen.** Users can access the system from a central page (http://huvariome.erasmusmc.nl) in which a genome is chosen and variants to be searched are uploaded as tab or space delimited search requests such that there is one variation per line. A region is searched by including an end position with the chromosome and start position.

**Figure 3:**
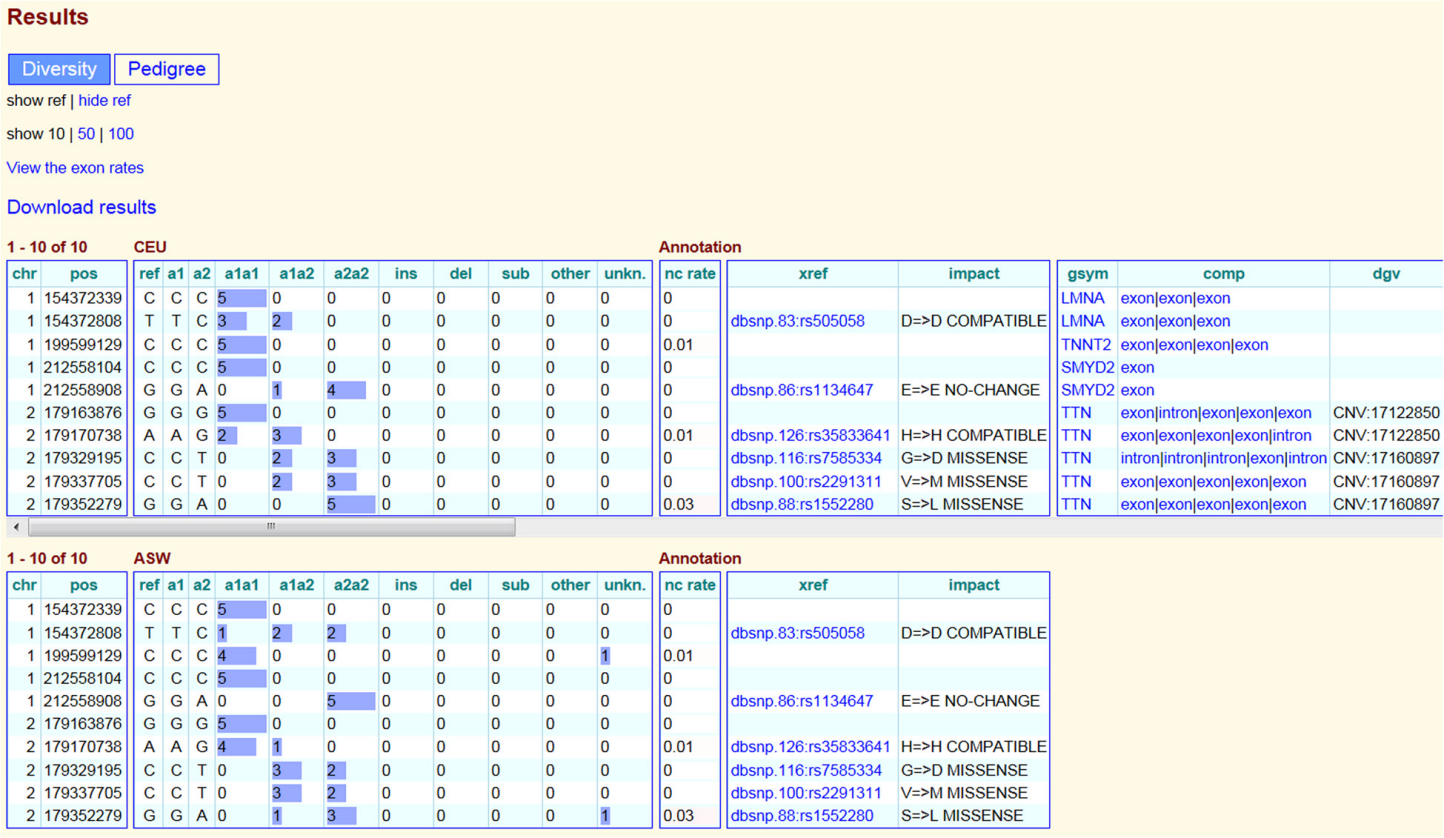
**Huvariome genotype frequency page.** The output page giving the distribution of allelic variations in the Diversity Panel of genomes for the European (CEU) and African (ASW) populations. In this example the first ten positions that were queried from the cardiomyopathy data set from Meder *et al*. 2011 [25] are shown. Each variant is returned per row with the frequency of each genotype highlighted by the size of the associated blue bar. Abbreviations: chromosome (chr); 0-based location (pos); reference allele (ref); variant alleles 1 and 2 (a1, a2); indels (ins, del); substitutions (sub); no-call (unkn.); no-call rate (nc rate); external reference (xref); predicted amino acid change (impact); gene symbol (gsym); gene component (comp), e.g. exon, intron; and variant from database of genomic variants (dgv).

Data in Huvariome can be queried and retrieved through a web interface that allows users to search the datasets for a specific gene or request information for a genomic region by means of a list of positions. The user inputs variations as a tab or space separated list of variation positions in the format chromosome, begin, end (optional) using a zero based format [18] (Figure 2). After the query is completed, the results, e.g. a table of allelic frequencies ordered by position per genetic group in the Diversity Panel, are returned on a webpage via an email containing the link to the table. Results for Huvariome Core (HVC) include the SNV quality measures derived from HVC and the Diversity Panel and are annotated with functional impact derived from Complete Genomics annotation. The results are additionally annotated with existing genetic variations, regulation features and disease association, as mentioned above. Registered users can submit a list of genomic positions to obtain allele frequencies and corresponding annotation for each of the HVC, the Diversity Panel, a Disease Cohort (genomes with associated disease phenotype), and a Pedigree Cohort (genomes of related individuals). Guest users can submit a list of genomic positions to receive the allele frequency for up to fifty nucleotide positions from the Diversity Panel with the associated no-call rate (see section Allele No-call rate). Common annotation, symbol, transcript identifier, the impact on the gene (gene component), known genomic variations (DGV), and regions of common sequences in multiple species (VISTA) are provided for each variation (Figure 3). Variations specific to a population, such as with the Diversity and Pedigree Panels, are returned as population specific variations which include the impact of variation on the coding sequence and associated dbSNP variants (Figure 3). Users can submit a list of genomic positions to receive the allele frequency for up to one hundred nucleotide positions and corresponding annotation for the Diversity Panel, the Pedigree Cohorts, the associated common variation tag (without frequencies from the HVC Panel) and the associated no-call rate. This functionality is accomplished by storing each observed variation indexed by both the library to which they belong and the location of origin in the genome to which the sequences were aligned. Registered users for can access their own genomes for study from the same access page.

## Utility

### Content and individual characteristics

The Huvariome database currently contains variations from 165 deep sequenced human genomes, at the time of writing, obtained with WGS, including tumor and associated normal genomes, small pedigrees and 42 individuals representing 10 different populations. The 69 individual freely available genomes [19] are non-diseased samples which include a Yoruban trio, a CEPH/Utah pedigree of 17 family members, a Puerto Rican trio, and a diversity panel representing nine different populations, from the Coriell Institute for Medical Research. Samples were sequenced to an average genome-wide coverage of about 80X (range of 51X to 89X). Huvariome includes the additional control dataset, beyond the Diversity and Pedigree Panel, called Huvariome Core (HVC), which is a subset of 31 genomes from unrelated individuals with no detectable disease association. The average coverage for HVC genomes is 70X with no-call rate of 3.5% and 3.25 million SNVs per individual genome (Table 2). Additional quality control analysis includes the calculation of the transition (Ti) to transversion (Tv) and heterozygous (hetero) to homozygous (homo) SNV ratios for several genomics features including, coding, intergenic, intronic, untranslated region, splice sites, transcription start site and the impact on the resultant protein as synonymous, missense and nonsense changes (Table 3). The Ti/Tv ratios for synonymous, missense and nonsense variants are 5.20, 2.14 and 1.93, respectively, in the 31 genomes which are in agreement with values of 5.6, 2.31 and 2.13 determined by Tennessen *et al.* 2012 [20]. The coding and intergenic Ti/Tv for the 31 genomes are 2.99 and 2.07, which are consistent with those published by Guo *et al.* 2012 [21] of 2.81 and 2.27 for exonic and non-exonic regions. The het/homo ratio for the same regions for HVC, ranges from 1.40-0.97 (Table 3) and are consistent with previous studies [20].

**Table 3:** Quality measures for huvariome genomes

**FEATURE**	**Ti/Tv**	**Hetero/homo**
CDS	2.99	1.13
INTERGENIC	2.07	1.15
INTRON	2.22	1.15
UTR	2.15	1.17
DONOR	2.81	0.97
TSS	2.08	1.16
ACCEPTOR	2.42	1.21
SYNONYMOUS	5.20	1.40
MISSENSE	2.14	1.28
NONSENSE	1.93	1.37

The transition (ti) to transversion (tv) and heterozygous (hetero) to homozygous (homo) ratios for SNVs were calculated for several regions of the genome, coding regions (CDS), intergenic, intronic (INTRON), untranslated region (UTR), splice sites (DONOR and ACCEPTOR), transcription region (TSS), and for different impact on the resultant protein sequence, no change in sequence (SYNONYMOUS), a change in the protein sequence with no change in size of protein (MISSENSE) and an early termination of protein translation (NONSENSE).

#### Allele no-call rate

We used the database to determine the no-call rate of allele calling at all 3 billion positions in the human genome. The control genomes are used to calculate a SNV no-call rate (nc rate) at the base pair level:

$$nc\;rate=1-\left(\frac{n}{t}\right)$$

where *n* is the number of no-calls (unidentifiable alleles at the position) and *t* is the total number of genomes. The fraction *n/t* is the proportion of alleles that are not able to be sequenced at a given base, and it is subtracted from 1 so that the higher the nc rate, the more plausible the base is called the correct nucleotide. In other words, this value indicates how likely the base is able to be sequenced and can be viewed as a measure of reliability for the individual base (Figure 3).

### Common variants

**Figure 4:**
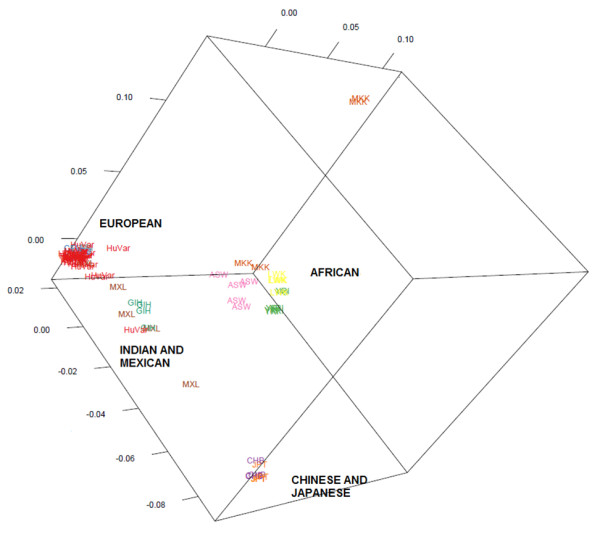
**Similarity of HVC to diversity panel.** The output from AWClust [23] is shown in a multi-dimensional scaling plot. The genomes of HVC samples (HuVar), the Chinese and Japanese (CHB, JPT), the African (MKK, ASW, YRI, LWK), the Indian and Mexican (MXL, GIH) and the European (CEU) populations are shown in this plot. The HVC (HuVar =red) overlap with the CEU population, indicating the samples are more similar to the European samples than the others.

The minor allele frequency (MAF) for each SNV in HVC of 31 genomes is calculated as the smaller of the number of occurrences of a reference allele or its variant allele divided by the number of samples (n=31) as outline by Zhu *et al.* 2011 [22]. A SNV with a MAF equal to zero indicates the genotype is the same for all samples and is subsequently removed whilst the remaining SNVs are placed into one of 31 MAF bins. The AWclust package [23] from Bioconductor, which is not included in Huvariome, was used to determine the similarity of the all genomes to the HVC samples using a modified input to match the application. The HVC samples (red) clearly segregate with the CEU and apart from the African and Asian populations of the Diversity Panel (Figure 4). The % of the number of SNVs with a MAF ≤ 5% or > 5% are 91.8% and 8.2% for the Exome Project and 87.4% and 12.4% for the 31 genomes used in Huvariome, providing evidence of consistency between these CEU study cohorts.

### Case study 1: confirmation of polymorphic variation

To identify new polymorphic variants and to demonstrate how Huvariome can provide an accurate prediction of rare SNVs we selected a set of 26 non-synonymous coding SNVs (cSNVs) which were found in one of the eight genomes sequenced in the study by Ng *et al*. 2009 [24]. Twenty six cSNVs which were called as potentially damaging but have ambiguous genotype information at these positions (Table 4, [24]) have been analyzed using Huvariome reference genomes, HVC and Diversity Panel (Table 2). The genotype for the eight HapMap samples (International HapMap Consortium, 2003) used in the study are displayed in the columns beginning with “NA” and the last three columns represent the genotypes predicted for each group from the two reference cohorts in Huvariome (Table 4). Seven of these 26 cSNVs genotypes are homozygous reference, as determined from Huvariome and heterozygous in the remaining 19 cases; the correct allele has been called in all cases (Table 4). This example demonstrates the value of a high quality reference cohort to help disambiguate potential false positive calls from NGS studies.

**Table 4:** Confirmation of genotypes from 26 ambiguous variations calls

					**European**		**African**				**Asian**			**Huvariome**		
**Chromosome**	**Position**	**Reference base**	**Amino acid change**	**Gene**	**NA12156**	**NA12878**	**NA18507**	**NA18517**	**NA19129**	**NA19240**	**NA18555**	**NA18956**	**Change**	**European**	**African**	**Asian**
2	227829345	G	ASP,TYR	COL4A3	**K**	G	G	G	G	G	G	G	**K**	G:T		G:T
6	71029714	T	GLU,GLY	COL9A1	**Y**	T	T	T	T	T	T	T	**Y**	**T:T**	**T:T**	**T:T**
14	70059717	T	TYR,CYS	ADAM20	**Y**	T	T	T	T	T	T	T	**Y**	**T:T**	**T:T**	**T:T**
16	28511156	T	ASN,THR	SULT1A2	**K**	T	T	T	T	T	T	T	**K**	T:G	T:G	T:G
9	100837151	C	PRO,LEU	COL15A1	C	**Y**	C	C	C	C	C	C	**Y**	C:C		
14	69994396	T	TYR,HIS	ADAM21	T	**Y**	T	T	T	T	T	T	**Y**	**T:T**	**T:T**	**T:T**
16	28514697	G	PRO,LEU	SULT1A2	G	**R**	G	G	G	G	G	G	**R**	G:A	G:A	
2	227632472	G	PRO,LEU	COL4A4	G	G	**R**	G	G	G	G	G	**R**		G:A	
2	237945216	G	ARG,TRP	COL6A3	G	G	**R**	G	G	G	G	G	**R**		G:A	
6	32286548	C	GLY,ARG	NOTCH4	C	C	**Y**	C	C	C	C	C	**Y**		C:T	
10	123233227	C	ARG,GLN	FGFR2	C	C	**Y**	C	C	C	C	C	**Y**	**C:C**	**C:C**	**C:C**
15	72802259	A	ILE,THR	CYP1A1	A	A	**R**	A	A	A	A	A	**R**		A:G	
15	82491107	T	MET,THR	ADAMTSL3	T	T	**Y**	T	T	T	T	T	**Y**		T:C	
19	46210061	T	ILE,THR	CYP2B6	T	T	**Y**	T	T	T	T	T	**Y**	**T:T**	**T:T**	**T:T**
4	73407648	C	GLY,ARG	ADAMTS3	C	C	C	**Y**	C	C	C	C	**Y**		C:T	
6	46728211	C	ARG,PRO	CYP39A1	C	C	C	**S**	C	C	C	C	**S**	C:G	C:G	
7	99283117	G	ARG,GLN	CYP3A43	G	G	G	**R**	G	G	G	G	**R**		G:A	
10	96474119	G	VAL,LEU	CYP2C18	G	G	G	**K**	G	G	G	G	**K**		G:T	
10	96698964	A	HIS,ARG	CYP2C9	A	A	A	**R**	A	A	A	A	**R**		A:G	
15	76845583	G	PRO,LEU	ADAMTS7	G	G	G	**R**	G	G	G	G	**R**		G:A	
1	120269806	C	GLY,ARG	NOTCH2	C	C	C	C	C	**Y**	C	C	**Y**	**C:C**	**C:C**	**C:C**
8	24420887	C	PRO,LEU	ADAM7	C	C	C	C	C	**Y**	C	C	**Y**	**C:C**	**C:C**	**C:C**
17	38961570	G	PRO,LEU	ETV4	G	G	G	G	G	G	**R**	G	**R**			G:A
21	46365928	C	PRO,THR	COL6A2	C	C	C	C	C	C	**M**	C	**M**			C:A
2	189622367	G	PRO,LEU	COL5A2	G	G	G	G	G	G	G	**R**	**R**			G:A
5	129100767	C	THR,ILE	ADAMTS19	C	C	C	C	C	C	C	**T**	**T**		C:T	

Results from Huvariome analysis of 26 selected coding SNVs to disambiguate genetic variation determined by Ng *et al.* 2009 [24]. The first 7 columns display the normal variations and their proposed functional impact, determined by Ng *et al*. 2009. The base changes are presented as the IUPAC codes per sample (e.g. NA12156), which are grouped by populations, CEU (NA12156, NA12878), YRI (NA18507, NA18517, NA19129, NA19240), Asian (NA18555, NA18956), with the impacted bases denoted with bold letters, and in the column titled “change”. The last three columns contain the genotypes called for the three populations present in Huvariome Core and the Diversity Panel (European, African, and Asian). The Huvariome genotypes highlighted as bold demonstrate that Huvariome calls homozygous reference while the genotypes are heterozygous reference.

### Case study 2: cardiomyopathy genes

In this example we use the recently described list of resequenced candidate cardiomyopathy associated genes [25] to determine the common variants and the non-polymorphic variants, and therefore candidate cardiomyopathy genes, in our cohorts. A list of 38 randomly selected variants and 6 variations associated with DCM or HCM which were Sanger sequenced to confirm their presence in the sample of origin (Table 5 and 5[25]), were analyzed for allele frequency variation using Huvariome. Meder *et al.* 2011 [25] could confirm 86% of variants by Sanger sequencing and we could predict normal alleles in 100% of cases with an nc rate (0.0-0.1) based on the two reference cohorts, Diversity Panel and HVC. The 30 variants, present in 19 genes, were confirmed in Huvariome as being polymorphic with 26 having an associated dbSNP number and two of the polymorphic variants not being present in HVC genomes (Table 5). The six known variants were annotated in Huvariome (Table 6, HGMD) with Biobase HGMD and confirmed the prediction, however the reverse complement is listed if in Huvariome the gene in HGMD is on the reverse strand (e.g. MYBP3 47324447C>T is listed as 977G>A in Table 6). An additional microdeletion in TNNT2 is present in HGMD whereas deletion of C was originally observed (Table 6) [25]. In addition to the six known cardiomyopathy variants there are six novel candidate caridomyopathy variants for which the genomic positions are homozygous in all 71 reference genomes (Table 6).

**Table 5:** Confirmation of known population variation

**Gene symbol**	**Chromosome**	**Reference position**	**Reference allele**	**Variant allele**	**Confirmed by sanger sequencing**	**Huvariome alleles**	**dbSNP ID**
LMNA	1	154372809	T	C	Yes	T/C	dbsnp.83:rs505058
SMYD2	1	212558909	G	A	Yes	G/A	dbsnp.86:rs1134647
TTN	2	179163877	G	A	Yes	G/A	dbsnp.130:rs72646845
TTN	2	179170739	A	G	Yes	A/G	dbsnp.126:rs35833641
TTN	2	179329196	C	T	Yes	C/T	dbsnp.116:rs7585334
TTN	2	179337706	C	T	Yes	C/T	dbsnp.100:rs2291311
TTN	2	179352280	G	A	Yes	G/A	dbsnp.88:rs1552280
HDAC2	6	114372280	C	**T**	Yes	T/C	dbsnp.121:rs13204445
TMEM2	9	73549916	C	T	Yes	C/T	dbsnp.72:rs25689
TMEM2	9	73550029	C	**G**	Yes	G/C	dbsnp.107:rs3739783
MYPN	10	69603927	G	A	No	G/A	dbsnp.120:rs10997975
LDB3	10	88483707	A	T	Yes	A/T	dbsnp.127:rs45567939
TRAF6	11	36473064	T	C	Yes	T/C*	
MYBPC3	11	47326019	G	A	Yes	G/A	dbsnp.120:rs11570058
MYBPC3	11	47326617	T	C	Yes	T/C	dbsnp.107:rs3729989
MYH6	14	22931651	A	G	Yes	A/G	dbsnp.80:rs365990
MYH7	14	22968900	G	A	No	G/A	dbsnp.86:rs735712
DICER1	14	94626500	A	T	Yes	A/T	dbsnp.52:rs13078
ACTC1	15	32868460	G	C	Yes	G/C	dbsnp.116:rs8037241
TPM1	15	61138893	C	A	Yes	C/A	dbsnp.86:rs1071646
TCAP1	17	35075837	A	C	Yes	A/C	dbsnp.86:rs1053651
DSC2	18	26903040	T	C	Yes	T/C**	
DSG2	18	27365107	G	A	Yes	G/A	
DSG2	18	27376616	G	A	Yes	G/A	
TNNI3	19	60357396	A	C	Yes	A/C	dbsnp.116:rs7252610
PARVB	22	42726784	T	C	Yes	T/C	dbsnp.86:rs1007863
PARVB	22	42821201	T	C	Yes	T/C	dbsnp.92:rs1983609
PARVB	22	42821229	T	C	Yes	T/C	dbsnp.86:rs738479
DMD	X	31406271	C	T	Yes	C/T	dbsnp.89:rs1800280
DMD	X	32413115	T	C	Yes	T/C	dbsnp.79:rs228406

Genomic nucleotide positions 1-based (Reference Position), nucleotides (Reference and Variant Alleles), and Confirmation by Sanger Sequencing are determined by Meder *et al.* 2011 [25]. The Variant Alleles in bold are the reference alleles in NCBI build 36. Huvariome Alleles are represented with the NCBI build 36 reference allele first in the pair (e.g. T/C with T from NCBI build 36). The T/C variant labeled with * is not found in the HVC, but in the CEU and GIH population; the T/C variant labeled with ** is not found in the HVC, but in the YRI and JPT population.

**Table 6:** Variations in candidate cardiomyopathy genes

**Gene symbol**	**Chromosome**	**Reference position**	**AA exchange**	**Reference allele**	**Variant allele**	**Confirmed by sanger sequencing**	**Huvariome alleles**	**Known pathological variant**	**Gene strand**	**HGMD**
LMNA	1	154372340	R>Stop	C	T	Yes	C/C	R321ter	+	Cardiomyopathy,_dilated|961C>T
TNNT2	1	199599130	E163fs	C	--	No	C/C		--	Cardiomyopathy,_hypertrophic|487G>A
SMYD2	1	212558105	H>Y	C	T	Yes	C/C			
DSP	6	7525794	R>G	C	G	No	C/C			Arrhythmogenic_right_ventricular_dysplasia/ cardiomyopathy|4372C>G
TMEM2	9	73505380	T>T	C	T	Yes	C/C			
ILK	11	6585971	P>L	C	T	No	C/C			**Cardiomyopathy,_dilated|209C>T**
MYBPC3	11	47324447	R>Q	C	T	Yes	C/C	R326Q	--	**Cardiomyopathy,_hypertrophic|977G>A**
MYBPC3	11	47313209-47313210	P955fs	CT	--	Yes	AG/AG	P955fs		Cardiomyopathy,_hypertrophic|2864_2865delCT
MYBPC3	11	47321263-47321264	F412fs	TT	--	Yes	AA/AA	F412fs		Cardiomyopathy,_hypertrophic|1235_1236delTT
MYH7	14	22963165	C905fs	G	--	No	A/A			
MYH7	14	22968054	R>C	G	A	Yes	G/G	R453C	--	Cardiomyopathy,_hypertrophic|1357C>A
MYH7	14	22971706	Y>H	A	G	Yes	A/A			**Cardiomyopathy,_hypertrophic|484T>C**
MYH7	14	22971762	R>Q	C	T	Yes	C/C	R143Q	--	Cardiomyopathy,_hypertrophic|428G>A

Genomic nucleotide positions 1-based (Reference Position), nucleotides (Reference and Variant Alleles), and Confirmation by Sanger Sequencing are determined by Meder *et al.* 2011 [25]. Huvariome alleles are represented with the NCBI build 36 reference allele first in the pair (e.g. T/C with T from NCBI build 36). Variants that have previously been found to be associated with cardiomyopathy are denoted by Known Pathological Variant [25] and cardiomyopathy variations derived from the professional edition of Human Gene Mutation Database (HGMD) were supplied by Biobase. The HGMD descriptions in **bold** are linked to the first being described by Meder *et al.* 2011 [25] as related to dilated or hypertrophic cardiomyopathy.

## Common gene single nucleotide variation rate

In addition to the allele frequencies from HVC, Diversity and Pedigree Panels, any variant which is known to be part of a gene is used to search our database germ line SNV reported for the HVC reference set. We implemented a method to calculate the exon variation rate *r* per gene:

$$r=-\text{ln}\left(\sum_{i=1}^{m}\frac{v_{i}}{\sum_{i=1}^{m}l_{i}}\right)$$

Where *m* is the number of exons within a given gene,*v*_*i*_ is the number of variants in exon *i*, and *l*_*i*_ is the length (in base pairs) of exon *i*. The ratio within parentheses is the proportion of bases that are variants in exons out of all bases within the exons of the gene. The negative log transformation produces a score such that a relatively small value corresponds to a gene with a large number of variants per base within the exon. Likewise, a larger score indicates a gene with a smaller number of variants per base within the exon. The nine candidate cardiomyopathy genes from Table 6 were used to search this resource. The results (Table 7) demonstrate that these genes have similar mutation rates compared with all known genes (26,000) listed in the database where the most variable gene is HLA-DRB6 (rate = 2.1) and the least is AHNAK (rate = 9.8). These data suggest that these nine genes are correctly annotated with no missed paralogs and add support that these variations (Table 6) are associated with cardiomyopathy candidate genes.

**Table 7:** Rate of reference genome variation in candidate cardiomyopathy genes

**Gene symbol**	**Ref var count**	**Exon length (bp)**	**Rate (r)**	**Var count**
TNNT2	2	1153	6.4	1
LMNA	5	3225	6.5	1
SMYD2	2	1685	6.7	1
TMEM2	6	6523	7.0	1
MYH7	5	6030	7.1	4
DSP	7	9730	7.2	1
ILK	1	1797	7.5	1
MYBPC3	2	4218	7.7	5

This table displays the number of variations in the genes that are used for searching Huvariome (Var count), the expected number of variants in the 31 genomes of HVC (Ref var count), the cumulative exon length in bp and the resultant rate of variants within the exons (negative log).

## Discussion

Huvariome was developed utilizing Oracle 11i technology designed to run on the Oracle Exadata platform [26], which was selected based on a number of favorable characteristics including scalability (Exadata scales linearly with added hardware) and performance (smart scans and hybrid columnar compression providing deep compression) [27]. This ensures that data do not need to be replicated as in a de-normalized data delivery platform such as Biomart [28] in which the data in the primary tables must be transformed and thus replicated to deliver fast return of results.

WGS was chosen as a basis for Huvariome to provide research scientists and the research community with a reference cohort for allele frequencies and for base quality checking at any position in the human genome. Here the database is presented as a resource for prioritizing rare SNVs identified with NGS technology. In contrast to other projects, only high coverage genome sequences are used and no imputation has been performed to infer unsequenced variants. Huvariome has been successfully used to prioritize candidate cancer targets and genomic variations detected in familial congenital malformations [29].

The system has been developed to address the need to access genetic variation frequency and assigned probability in control population datasets (e.g. to determine the frequency of a change in the population) and to perform aggregate analyses and assign validation probabilities to observed, naturally occurring variants based on sequencing characteristics across a population. To support these goals we have included the common variation determined in a reference population representative of the Benelux population as part of the output from the public reference datasets provided. In addition we have used the common variations present in HuVariome Core and the Diversity panel to determine the allele “no-call” rate per base of the human genome. We have demonstrated the ability of Huvariome to determine the variants in another resequencing project [24] and to support candidate gene selection in a cardiomyopathy resquencing project [25].

## Conclusions

Huvariome was developed to facilitate data storage of WGS and the analysis of genetic variation detected by WGS in research and clinical diagnostics environment, which both require a secure and scalable database. Huvariome provides a user-friendly interface to access genetic variation data from diverse cohort studies for the identification of disease-promoting variations in the underlying database. The variants are annotated to provide users with a wealth of information that they would otherwise have to retrieve manually. The use of high depth and low error whole genome sequencing ensures a high accuracy of allele calling, and the no-call rate offers additional information about the allele frequencies at each base in the human genome build. The database is currently used for several tasks including SNV discovery and *in silico* validation. Since Huvariome contains data from experiments as well as from reference cohorts, we can separate rare polymorphisms from candidate disease-causing variants. Access to variations obtained from the public Diversity Panel data is freely available from the Huvariome web site. The examples show that Huvariome is a powerful application to confirm ambiguous genotype calls with the associated no-call per base of the human genome. The application allows users to easily compare their genotypes with the 69 reference genomes of the Diversity Panel and Pedigrees to prioritize the candidate gene selection for both family and tumor-based genome analysis. The use of Huvariome Core samples provide additional support to determine if a variant is common (or rare), if the gene that is a candidate has an excess of variations beyond what is statistically expected if a variant is common, and the no-call rate associated with sequencing any base in the reference genome. This application has been successfully used in candidate gene selection for both tumor profiling and Mendelian inheritance studies [29].

We are currently enhancing the performance and scalability by migrating this application to run on Oracle Exadata hardware, allowing highly optimized parallel processing and high compression capability for cheaper storage and faster querying [27], and are developing summary pages that include visualizations using TIBCO Spotfire Web Player technology [30]. The data loaders developed in this project can easily be adapted to accommodate changes in data format thereby making the database sequencing platform independent which will allow sequencing results from other NGS platform (e.g. Illumina, Roche, Life Technologies) and data types (e.g. RNAseq) to be incorporated into this database.

We encourage collaborators to upload their own variants files into the knowledge archive initially in collaboration with the Erasmus University Medical Center and in the future via an optimized upload website with an agreed policy and standardized format and to ensure that the data quality is maintained.

### Availability

Huvariome is freely accessible for use from the web site at URL: http://huvariome.erasmusmc.nl.

## Abbreviations

SNV: Single nucleotide variation; HVC: Huvariome Core; WGS: Whole genome sequence; MAF: Minor allele frequency; DGV: Database of genomic variants; NGS: Next generation sequencing.

## Competing interests

The authors declare that they have no competing interests.

## Authors’ contributions

AS, EM, DL, PvdS and AHJK drafted the manuscript. SH, IP, AHJK and AS are involved with the design, data integration and maintenance of the database and application. EM, JR, SDH and AS are responsible for the case studies and statistical analysis. SS, DH, SN, AK, JM and PvdS provided analytical support and testing of the application. All authors read and approved the final manuscript.
